# Metabolomic and Lipidomic Analysis of Serum Samples following *Curcuma longa* Extract Supplementation in High-Fructose and Saturated Fat Fed Rats

**DOI:** 10.1371/journal.pone.0135948

**Published:** 2015-08-19

**Authors:** Fabrice Tranchida, Laetitia Shintu, Zo Rakotoniaina, Léopold Tchiakpe, Valérie Deyris, Abel Hiol, Stefano Caldarelli

**Affiliations:** 1 Aix Marseille Université, Centrale Marseille, Centre National de Recherche Scientifique (CNRS), Institut des Sciences Moléculaires de Marseille (iSm2) Unité Mixte de Recherche (UMR) 7313, Marseille, France; 2 Aix-Marseille Université, Laboratoire de Nutrition-Diététique, Faculté de Pharmacie, Marseille, France; 3 Centre de coopération internationale en recherche agronomique pour le développement (CIRAD), Unité Mixte de Recherche (UMR) QualiSud, Université de La Réunion, Ecole Supérieure d’Ingénieurs Réunion Océan Indien (ESIROI), Saint Denis, France; 4 Institut de Chimie des Substances Naturelles, Unité Propre de Recherche (UPR) 2301, Centre National de Recherche Scientifique (CNRS), Gif-sur-Yvette, France; Bambino Gesu' Children Hospital, ITALY

## Abstract

We explored, using nuclear magnetic resonance (NMR) metabolomics and fatty acids profiling, the effects of a common nutritional complement, *Curcuma longa*, at a nutritionally relevant dose with human use, administered in conjunction with an unbalanced diet. Indeed, traditional food supplements have been long used to counter metabolic impairments induced by unbalanced diets. Here, rats were fed either a standard diet, a high level of fructose and saturated fatty acid (HFS) diet, a diet common to western countries and that certainly contributes to the epidemic of insulin resistance (IR) syndrome, or a HFS diet with a *Curcuma longa* extract (1% of curcuminoids in the extract) for ten weeks. Orthogonal projections to latent structures discriminant analysis (OPLS-DA) on the serum NMR profiles and fatty acid composition (determined by GC/MS) showed a clear discrimination between HFS groups and controls. This discrimination involved metabolites such as glucose, amino acids, pyruvate, creatine, phosphocholine/glycerophosphocholine, ketone bodies and glycoproteins as well as an increase of monounsaturated fatty acids (MUFAs) and a decrease of n-6 and n-3 polyunsaturated fatty acids (PUFAs). Although the administration of *Curcuma longa* did not prevent the observed increase of glucose, triglycerides, cholesterol and insulin levels, discriminating metabolites were observed between groups fed HFS alone or with addition of a *Curcuma longa* extract, namely some MUFA and n-3 PUFA, glycoproteins, glutamine, and methanol, suggesting that curcuminoids may act respectively on the fatty acid metabolism, the hexosamine biosynthesis pathway and alcohol oxidation. *Curcuma longa* extract supplementation appears to be beneficial in these metabolic pathways in rats. This metabolomic approach highlights important serum metabolites that could help in understanding further the metabolic mechanisms leading to IR.

## Introduction

Fructose consumption from corn syrup, a common sweetener used in the food industry, has increased dramatically over the past few decades in industrialized countries, and its impact on health has been recently reviewed [1]. Similarly, the intake of saturated fats has risen during the same time period. It has been reported that these two factors contribute to the epidemic of metabolic syndrome [2,3], which is generally considered to be an association of impaired glucose tolerance, hypertension, dyslipidemia, hyperuricemia and central obesity in human beings and animals [4]. Many studies have shown that insulin resistance (IR) is directly associated with lipid disorders, which induced alterations of insulin action and signalling pathways [5]. Moreover, model animals fed a high fructose and high fat diet experienced an increased production of reactive oxygen species (ROS) and/or reactive nitrogen species (RNS) with impaired antioxidant defences [6]. As a consequence, an imbalance between reactive molecular species and antioxidant defences was observed in the development of insulin resistance, impaired insulin secretion and during late complications of diabetes [7].

During the last decade, the development as metabolic disorder treatments of traditional medicine based on natural products has dramatically increased. In this paper, we were particularly interested in the medicinal potential of *Curcuma longa* (CL), a perennial herb native to southern and southeastern tropical Asia commonly termed as turmeric (Zingiberaceae family). Indeed, CL is widely consumed in these regions as a dietary spice and food-coloring as well as for the prevention and therapy of various illnesses [8]. Despite their low bioavailability, curcuminoids, a group of phenolic compounds that are the major bioactive constituent of turmeric extracts, have been shown to possess helpful antioxidant, anticarcinogenic, anti-inflammatory, hypoglycemic, and hypolipidemic actions in animal models as well as human clinical trials [9]. Furthermore, in rats, curcumin, the major curcuminoid present in turmeric, ameliorates IR and diabetes by increasing the uptake and oxidation of fatty acids and glucose in skeletal muscle [10]. However, all these studies were usually performed using concentrations of curcuminoids much higher than those used in nutritional supplements. Indeed, the effect of these supplements taken at a lower dose has been rarely explored yet [11]. In order to characterize its effects on the metabolism, we chose to analyze serum samples from rat fed diets with high fructose and saturated fatty acids alone (HFS) or with the addition of a curcuma extract (HFS+C) using metabolomic and biochemical approaches. Metabolomics has been successfully applied to highlight markers of metabolic alterations in plasma or serum from high-fat and/or high-carbohydrate (fructose and sucrose) fed rodents using nuclear magnetic resonance (NMR) [12,13], or liquid-chromatography coupled with mass spectrometry (LC-MS) [14]. Herein, metabolites and the FA affected by the HFS diet or the absorption of the curcuma extract were identified using NMR and GC/MS-based metabolomics and lipidomics, respectively. We also measured serum antioxidant capacity and lipid peroxidation in order to assess the oxidative stress level in each serum sample.

To the best of our knowledge, no previous study has used an NMR-based metabolomics approach to evaluate the metabolic consequences in response to exposure to HFS diet in rats in conjunction with an extract of CL to highlight possible beneficial effects of this latter.

## Materials and Methods

### Reagents

All chemicals used in this study were of the highest grade. Acetyl chloride, 2,6-di-tert-butylp-cresol, butylated hydroxytoluene (BHT), methanol, hexane, the internal (23:0 methyl ester), Trolox (6-hydroxy-2,5,7,8-tetramethyl-chroman-2-carboxyl acid) and AAPH (2,2’-azobis(2-amidinopropane)dihydrochloride) were purchased from Sigma-Aldrich (Sigma-Aldrich Chimie S.a.r.l, Lyon, France). BHT was added to methanol (50 μg BHT/ml methanol) to prevent fatty acid oxidation. The external standards (fatty acid methyl ester, FAME) were purchased from Supelco 37 component FAME mix (Sigma-Aldrich Chimie S.a.r.l, Lyon, France). The internal standard was dissolved in the methanol-BHT solution at a concentration of 100 μg/ml.

### Preparation of the hydro-alcoholic extract of *Curcuma longa*

Powder of the rhizome of CL was provided by Laco SARL (Marseille, France). For the extraction, the rhizome of CL was macerated with hot water (80°C) for 4 h, and the aqueous extract was evaporated under vacuum at 60°C. The rhizome residue was re-extracted with ethanol at 60°C during 2 h, filtered, and evaporated under vacuum. The final extract was a 1:1 mixture of the aqueous and alcohol extracts that was re-dissolved with 2% alcohol and then with 0.9% NaCl for the per os administration.

### Experimental animals and diets

All animal protocols were approved by the Animal Ethics Committee of the Faculty of Pharmacy of Aix-Marseille Université (Marseille, France) in agreement with the guidelines of the French Ministry of Food and Agriculture. Thirty young male Sprague Dawley (SD) rats (180–200 g) purchased from Elevage Janvier (Le Genest St Isle, France) were maintained in a temperature-and humidity-controlled environment and fed ad libitum. After one week of adaptation under feeding with standard diet (3.32 kcal/g, SAFE, Augy, France), rats were randomly divided into three groups to receive: (i) standard diet (Control group, n = 6); (ii) high fructose and saturated fatty acids diet (4.3 kcal/g SAFE, Augy, France, HFS group, n = 12); (iii) high fructose and saturated fatty acids diet and hydroalcoholic extract of curcuma 100 mg/kg body weight/day by oral gavage (4.3 kcal/g, HFS + C group, n = 12). Control and HFS groups received the same dose of vehicle (a turmeric free hydroalcoholic solution). The total concentration of curcuminoids in the curcuma extract was 1%, as determined by HPLC. Body weight was monitored weekly. After 10 weeks of treatment, the rats were fasted overnight, anesthetized under the mixture of ketamine and xylazine at 60 mg/kg and 5 mg/kg, respectively, and blood sample was collected from the vena cava. Rats were then euthanized by exsanguination. Samples were then centrifuged for 10 min at 1000 g and sera collected and divided into 2 aliquots prior to storage at –80°C until analysis. Livers were removed and weighed. The composition of each diet is reported in Table 1.

**Table 1:** Composition of the diets and fatty acid profile.

Constituents (g/100 g dry weight)	Control diet	HFS diet
**Protein**	19	19
**Methionine**	0.3	0.3
**Starch**	62	−
**Sucrose**	3	−
**Cellulose**	5	−
**Fructose**	−	61.7
**Minerals and vitamins**	7	7
**Choline**	0.04	0.04
**Lard**	−	12
**Soybean and fish lipid sources**	3.5	−
**Main fatty acids (% total fatty acids)**		
**16:0**	15.5	24.6
**18:0**	traces	13.8
**16:1n-7**	2.3	2.2
**18:1n-9**	25	37.9
**18:2n-6**	48	10.8
**18:3n-3**	0.5	0.8
**∑SFA**	17	40.6
**∑MUFA**	30	46.9
**∑PUFA**	53	12.5

∑ SFA = total saturated fatty acids, ∑ MUFA = total monounsaturated fatty acids, ∑ PUFA = total polyunsaturated fatty acids.

### Biochemical analysis of serum

Cholesterolemia, triglyceridemia, glycemia, were quantified by SELARL Laboratoire de Biologie Médicale C. Carboni (Aubagne, France). Insulin concentration was measured using a commercial kit (Eurobio, France). An estimate of IR was determined using the homeostasis model assessment index for insulin resistance (HOMA-IR), which was calculated using the formula [fasting insulin (μU/ml) X fasting glucose (mmol/l)/R]. In our study, R = 87.51 was determined empirically as the divisor producing an average HOMA-IR of 1 in control rats, analogous to the assumptions applied in the development of HOMA-IR in humans [15].

### Fatty acids determination by gas chromatography-mass spectrometry

The FAME procedure described previously [16] was applied, using a Thermo Scientific ITQ 700 (Thermo Fisher Scientific) gas chromatograph-ion trap spectroscopy equipped with PEG columns (30 m X 0.25 mm id., 0.25 μm thickness) (DB-FFAP Agilent Technologies, France. Data collection and processing were performed by means of XCALIBUR software, (version 2.0 Thermo Fisher Scientific). The relative amount of each fatty acid (% of total fatty acids) was determined by integrating the area under the peak and dividing the result by the total area for all the fatty acid peaks present in the sample.

### Estimation of desaturase activity

The product to substrate ratios of (18:1n-9/18:0) and (16:1n-7/16:0), (20:3n-6/18:2n-6), (20:4n-6/20:3n-6) ratios were used to estimate respectively the activities of desaturases Δ9, Δ6 and Δ5 [17].

### Quantification of lipid peroxydation and total antioxidant capacity of serum

Malondialdehyde (MDA) levels were measured by the thiobarbituric assay (TBA), and were taken as an index of lipid peroxidation in serum. The MDA-TBA reaction was carried out by mixing 50 μL of plasma or 50 μL standard solutions (MDA tetrabutylammonium from 0.05 to 1.5 μM) with 10 μ L of butylhydroxytoluene (BHT, 10 mM) and 450 μL of TBA solution (25 mM in phosphoric acid (0.3 M)–KOH pH 3.5). Then, the reaction mixtures were homogenized by vortex mixing, and placed into a water bath of 95°C for 30 min. Subsequently, the samples were cooled down on ice for 2 h. The supernatant was collected and injected for analysis on a Merck Hitachi/Lachrom HPLC system (L-7100 pump, L-7612 degasser, D-7000 interface and L-7455 diode array detector) using a Macherey-Nagel C18 150/4 (5μM) column. The sample solutions were analyzed using the following HPLC conditions: mobile phase of methanol/20 mM pH 6.9 potassium phosphate buffer (3:7, v/v). Samples were analyzed by isocratic elution at 45% mobile phase for 8.0 min. The fluorescence wavelengths used were 532 nm and 553 nm for excitation and emission respectively.

The total antioxidant capacity (TAC) of serum was measured in duplicate by using the oxygen radical absorbance capacity (ORAC) assay [18] on a microplate reader Infinite 200 (Tecan Group Ltd, Männedorf Switzerland).

### ^1^H NMR Spectroscopy

After the biochemical analysis, fifty-four serum aliquots (6 pairs for controls; 11 pairs for HFS group; 9 pairs + 2 independent aliquots for HFS +C group) were available for the NMR analysis. NMR samples were prepared using 200 μl of serum mixed with 400 μl of 0.9 g.L^-1^ saline solution (50% D2O/H2O (v/v)). All the 1D ^1^H NMR experiments were carried out at 300 K on a Bruker Avance spectrometer operating at 500 MHz for the ^1^H frequency using a 5-mm cryoprobe. A first set of spectra was collected using a solvent suppression pulse sequence based on the 1D nuclear Overhauser effect spectroscopy pulse sequence (Trd-90°-t1-90°-tm-90°-Taq) for water suppression (Trd = 2s, tm = 150 ms, t1 = 3 μs) 64 free induction decays (FID) of 32,768 data points were collected using a spectral width of 10 kHz with an acquisition time of 1.64 s. A second set of spectra was acquired on the same samples using a Carr-Purcell-Meiboom-Gill (CPMG) pulse sequence (Trd-90°-{*τ*-180°-*τ*}n-Taq) with presaturation of the water signal during a recycle delay of 2s, with *τ* = 400 μs and n = 200, for a total filter delay of 160 ms, and the same other parameters described above. The two sets of spectra are complementary since the fatty acid and macromolecule signals dominate the NOESY spectrum when the CPMG experiment attenuates these large signals to unveil the signals of minor metabolites.

For all the spectra, the FIDs were multiplied by an exponential weighting function corresponding to a line broadening of 0.3 Hz and zero-filled before Fourier transformation. NMR spectra were phased and baseline corrected manually and referenced to the glucose α-anomeric signal (δ = 5.23 ppm).

In order to assign the detected metabolites, ^1^H-^1^H TOCSY [19] (24 transients, 256 increments, mixing time of 80 ms) and ^1^H-^13^C HSQC [20] experiments (64 transients, 256 increments) were performed on one sample from each group.

### Data processing

The ^1^H 1D NMR spectra were directly exported to AMIX 3.8 software (Bruker Biospin GmbH, Karlshure, Germany) and divided into 0.001 ppm-width buckets. In order to remove the effects of possible variations in the water suppression efficiency, the region between 4.20 and 5.00 ppm was discarded as well as the signals at 3.86–3.89, 3.52–3.57, 3.42–3.46 and 1.11–1.15 ppm of propylene glycol, an anaesthetic component. The obtained NMR dataset (54 observations x 6508 buckets) was then normalised to the total spectrum intensity. In order to create a model that will explain the impact of the diets on the whole metabolism of the rat and highlight potential correlation between the metabolome (NMR data) and the lipidome (GC/MS data), we combined the NMR dataset and the relative quantification of fatty acid composition in an unique X-matrix (54 observations x 6529 buckets) that was then subjected to statistical analysis using the software Simca-P 12 (Umetrics, Umeå, Sweden). The relative quantification of the fatty acids was replicated in the X-matrix in order to match the duplication of the NMR spectra.

### Statistical analyses

The results of the biochemical analysis and the FA composition of serum are expressed as means ± Standard Error of the Mean (SEM). Unpaired Student’s t-test (if the distribution was normal) or nonparametric test Mann−Whitney U test (if the distribution was non-normal) were used for the biochemical analysis. Differences were considered significant for p- value < 0.05. The FA composition data for the serum were first analyzed using one-way analysis of ANOVA (for FA values with normal distribution) or Kruskal-Wallis test (for FA values with non-normal distribution), which provide specific information on whether a group mean is significantly different from the others. When statistically significant, these tests were followed by a multiple comparison test (post hoc) that uses critical values from Student's t-distribution after a Bonferroni adjustment in order to minimize the proportion of false positives in multiple comparisons. In our study, pairwise comparisons were performed using the adjusted significant level α* = 0.017, the critical values being given by $t_{\left(N-k,\;\alpha^{*}\;/2\right)}$ and $t_{\left(N-k,\;1-\alpha^{*}\;/2\right)}$ with N = 30 (number of observations) and k = 3 (number of groups). The statistical tests were calculated using Sigma Stat software version 3.11 (Systat Software Inc, San Jose, Calif. United States) and MATLAB v7.4 software (The MathWorks Inc., Natick, Massachusetts, United States) for the biochemical and FA composition data, respectively.

Principal component analysis (PCA) was applied to the X-matrix described above as well as to the X-matrix without replication of the data (matrix of 28 samples and 6529 variables). This method was used to detect intrinsic clusters and outliers within the datasets. The comparison of both PCA models, built with or without replication, was carried out in order to assess the effect of the replication of the fatty acid quantification on the statistical model. Since discrimination was not achieved using PCA, the X-matrix was analyzed using supervised OPLS-DA [21] in which we defined the Y-matrix as the matrix of sample classes, i.e. 0 for the controls, 1 for HFS group and 2 for HFS+C group. Model validation was performed by re-sampling it 999 times under the null hypothesis, that is to say generating models with a randomly permuted Y matrix. The quality of the model was assessed by monitoring changes in goodness-of-fit and predictive statistics, R2 and Q2, between the permuted and the original Y matrices.

## Results

### Body weight gain and biochemical analysis

After 10 weeks of diet the body weight of HFS, HFS+C and control rats was similar in agreement with previous researches [22,23] while a significant increase of relative liver weight was observed in both HFS groups. Compared to control, high fructose and saturated fatty acids diets were associated with a significant increase of glucose, triglycerides, cholesterol and insulin and HOMA-IR levels (Table 2).

**Table 2:** Results of serum biochemical analysis after 10 weeks of diet.

Group	Controls	HFS	HFS+C
**Body Weight** (g)	435.17 ± 20.74	438.42 ± 31.85	444.75 ± 50.78
**Relative liver weight**	0.0262 ± 0.0008	0.0310 ± 0.0010**	0.0334 ± 0.0016**
**HOMAR-IR**	1 ± 0.33	10.79 ± 1.89**	6.97 ± 2.05*
**Glucose** (g/l)	1.07 ± 0.18	1.82 ± 0.41*	1.80 ± 0.44*
**Insulin** (μg/l)	0.48 ± 0.13	3.34 ± 0.50*	2.22 ± 0.69*
**Triglycerides** (g/d)	0.435 ± 0.15	0.878 ± 0.22*	1.114 ± 0.36*
**Total cholesterol** (g/l)	0.60 ± 0.02	0.68 ± 0.04*	0.66 ± 0.06

Relative liver weight is defined as liver weight divided by body weight. Values are mean ± S.E.M (n = 6–12 rats/group).

*P < 0.05 vs. the control

**P < 0.01 vs. the control.

### Oxidative stress

ORAC and MDA assays were performed to appreciate the total antioxidant capacity and lipid peroxidation level of the sera. Our results showed a decrease in HFS and HFS+C groups of the ORAC score (-12.5 and -23% respectively) associated with an increase (more than 2 fold) of the MDA concentration (Table 3).

**Table 3:** Measurements of lipid peroxidation, total antioxidant capacity of serum after 10 weeks of diet.

Group	Controls	HFS	HFS+C
**MDA** ^**a**^ (μmol/l)	0.31 ± 0.10	0.80 ± 0.07*	0.81 ± 0.048*
**ORAC** ^**b**^ (μmol Trolox equivalent/l)	2763.6 ± 108.9	2456.4 ± 84.3*	2243.1 ± 103.8*

^a^ Malondialdehyde

^b^ oxygen radical absorbance capacity.

Values are mean ± S.E.M (n = 6–12 rats/group).

*p < 0.05 vs. the control.

### Serum fatty acid (FA) profile

In all experimental groups, the major plasma FA were: palmitic (16:0), palmitoleic (16:1n-7), stearic (18:0), oleic (18:1n-9), cis-vaccenic (18:1n-7), linoleic acid (18:2n-6), arachidonic acid (20:4n-6) and docosahexaenoic acid (22:6n-3 or DHA) (Table 4). The change in FA composition between the controls and the HFS group was fully described in a previous study [22]. Basically, it was observed in the HFS group an increased activity of the delta-9 (Δ9D) and delta-6-desaturase (Δ6D), and a decrease of the delta-5-desaturase (Δ5D), as seen by the 20:4n-6/20:3n-6 ratio, in addition to the depletion of n-6 polyunsaturated fatty acids (n-6 PUFA), 18:2n-6 and its elongation and desaturation product, 20:4n-6. In addition, HFS dietary loading caused a decrease of n-3 PUFA, alpha linolenic acid (18:3n-3, -70%), eicosopentaenoic acid (20:5n-3 or EPA, -33%) and 22:6n-3 (DHA, -33%). The presence of curcuma in the diet did not modify these tendencies. However, the comparison between HFS and HFS+C groups showed a significant decrease of n-3 PUFA, oleic (18:1n-9), myristoleic (14:1) and nervonic acid (24:1n-9) (-75%, -36% and -36%, respectively), associated with a relevant increase of 14:1n-5 and 18:1n-9 acids (more than 2 fold and +22%, respectively).

**Table 4:** Fatty acid composition of serum.

Fatty acid	Controls	HFS	HFS+C	p-value
**C12:0 Lauric acid**	0.20 ± 0.05	0.12 ± 0.01	0.12 ± 0.02	0.09
**C14:0 Myristic acid**	0.83 ± 0.03	0.80 ± 0.05	0.82 ± 0.07	0.94
**C14:1n-5 Myristoleic acid**	0.13 ± 0.05	0.15 ± 0.02	0.33 ± 0.06 ^a^	0.009
**C16:0 Palmitic acid**	19.07 ± 0.91	19.98 ± 0.25	19.97 ± 0.54	0.49
**C16:1n-7 Palmitoleic acid**	1.80 ± 0.09	2.60 ± 0.26	2.33 ± 0.38	0.24
**C18:0 Steric acid**	24.46 ± 0.87	21.71 ± 1.43	20.46 ± 1.91	0.34
**C18:1n-9 Oleic acid**	7.73 ± 0.20	17.64 ± 0.92 ^a^	21.48 ± 1.30 ^a^ ^,^ ^b^	0.00000011
**C18:1n-7 *cis*-Vaccenic acid**	2.37 ± 0.11	3.14 ± 0.23	2.77 ± 0.31	0.12
**C18:2n-6 Linoleic acid**	17.00 ± 0.61	13.09 ± 0.68 ^a^	13.26 ± 0.93	0.013
**C18:3n-6 gamma Linolenic acid**	0.11 ± 0.02	0.16 ± 0.03	0.16 ± 0.07	0.57
**C18:3n-3 alpha Linolenic acid**	0.40 ± 0.11	0.12 ± 0.02	0.09 ± 0.02 ^a^	0.003
**C20:0 Arachidic acid**	0.60 ± 0.13	0.40 ± 0.03	0.28 ± 0.06 ^a^	0.011
**C20:1n-9 Gondoic acid**	0.56 ± 0.12	0.12 ± 0.02 ^a^	0.17 ± 0.03	0.018
**C20:3n-6 Dihomo gamma linolenic acid**	0.12 ± 0.03	0.52 ± 0.06 ^a^	0.41 ± 0.04 ^a^	0.0002
**C20:4n-6 Arachidonic acid**	18.69 ± 1.09	15.02 ± 1.08	14.38 ± 0.76 ^a^	0.003
**C20:5n-3 Eicosopentaenoic acid**	0.30 ± 0.06	0.20 ± 0.03	0.05 ± 0.02 ^a^ ^,^ ^b^	0.0001
**C22:0 Behenic acid**	0.28 ± 0.06	0.23 ± 0.02	0.16 ± 0.03	0.05
**C22:4n-6 Adrenic acid**	0.86 ± 0.20	0.22 ± 0.02	0.11 ± 0.05 ^a^	0.004
**C24:0 Lignoceric acid**	0.86 ± 0.22	0.90 ± 0.10	0.80 ± 0.12	0.83
**C22:6n-3 Docosahexaenoic acid**	2.65 ± 0.29	1.77 ± 0.16 ^a^	1.14 ± 0.06 ^a^ ^,^ ^b^	0.00005
**C24:1n-9 Nervonic acid**	0.98 ± 0.27	1.11 ± 0.09	0.71 ± 0.07 ^b^	0.014
∑ **SFA**	46.30 ± 1.38	44.06 ± 1.50	42.58 ± 2.15	0.50
∑ **MUFA**	13.57 ± 0.41	24.73 ± 0.98 ^a^	27.79 ± 1.59 ^a^	0.0005
∑ **PUFA**	40.12 ± 1.37	31.21 ± 0.88 ^a^	29.60 ± 1.21 ^a^	0.0015
∑ **PUFA n-3**	3.35 ± 0.43	2.08 ± 0.15	1.29 ± 0.06 ^a^ ^,^ ^b^	0.000038
∑ **PUFA n-6**	36.78 ± 1.4	29.13 ± 0.81 ^a^	28.32 ± 1.22 ^a^	0.0042
Δ **9 C16:1n-7/C16:0**	0.09 ± 0.003	0.13 ± 0.01	0.12 ± 0.02	0.314
Δ **9 C18:1n-9/C18:0**	0.32 ± 0.007	0.87 ± 0.09 ^a^	1.23 ± 0.19 ^a^	0.0005
Δ **6 C20:3n-6/C18:2n-6**	0.01 ± 0.0005	0.04 ± 0.003 ^a^	0.03 ± 0.003 ^a^	0.0004
Δ **5 C20:4n-6/C20:3n-6**	128.67 ± 13.5	36.35 ± 6.16 ^a^	39.24 ± 5.09 ^a^	0.0008

Relative fatty acid composition (% of total) and estimated desaturase activities in serum of rats fed control, HFS and HFS+C diets. Values are mean ± S.E.M (n = 6–12 rats/group). Samples were measured in duplicate. ∑ SFA total saturated fatty acids, ∑ PUFA total polyunsaturated fatty acids, ∑ MUFA total monounsaturated fatty acids, Δ estimated desaturase activity.

^a^ significantly different from control group

^b^ significantly different from HFS group; after multiple comparison tests and Bonferroni adjustment of the significance level.

### ^1^H NMR spectroscopy of serum samples

**Fig 1:**
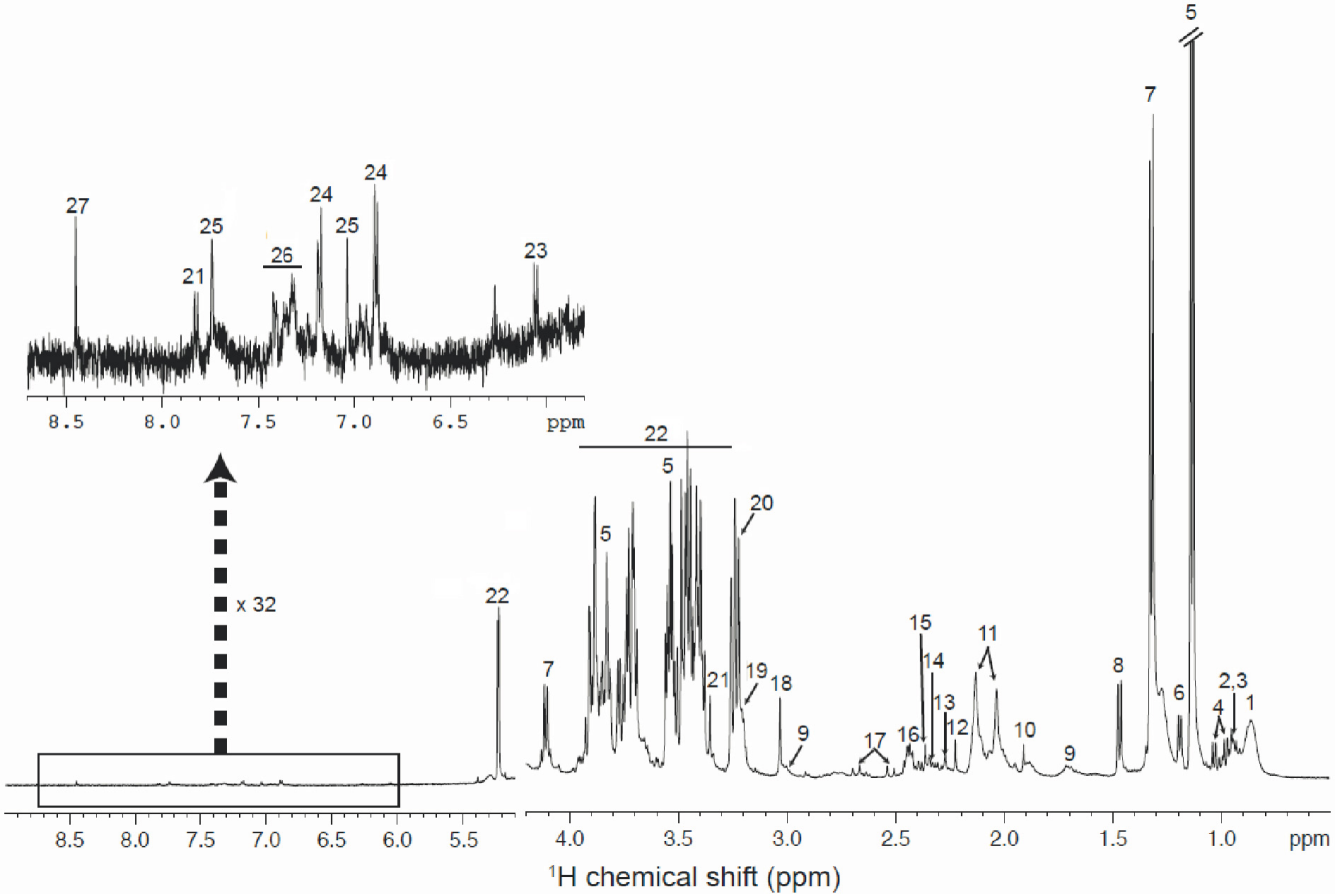
^1^H CPMG NMR spectrum of serum sample from rats fed with the HFS+C diet. Assignments: 1. lipids; 2. isoleucine; 3. leucine; 4. valine; 5. propylene glycol; 6. β-hydroxybutyrate; 7. lactate; 8. alanine; 9. lysine; 10. acetate; 11. glycoproteins (acetyl); 12. acetoacetate; 13. unknown; 14. glutamate; 15. pyruvate; 16. glutamine; 17. citrate; 18. creatine; 19. choline; 20. phosphocholine/glycerophosphocholine; 21. methanol; 22. alpha-glucose and beta-glucose; 23. cytidine; 24. tyrosine; 25. histidine; 26. phenylalanine; 27. formate.

A typical example of a ^1^H CPMG 1D NMR spectrum of a serum sample from a rat fed with a HFS diet supplemented with curcuma extract is shown in Fig 1 (see S1 Fig for an example of a NOESY 1D spectrum of the same sample) and assignments of metabolites are given in S1 Table.

The spectra of serum samples from HFS+C group are dominated by the signals of glucose and lactate, correlated with the hyperglycemia of the rat under high fructose and fat diet. Signals from propylene glycol, a component from the anaesthetic substance used before the sacrifice of the animals, were at a high concentration in the samples and were not included in the statistical analysis.

### Multivariate statistical analysis of serum samples

**Fig 2:**
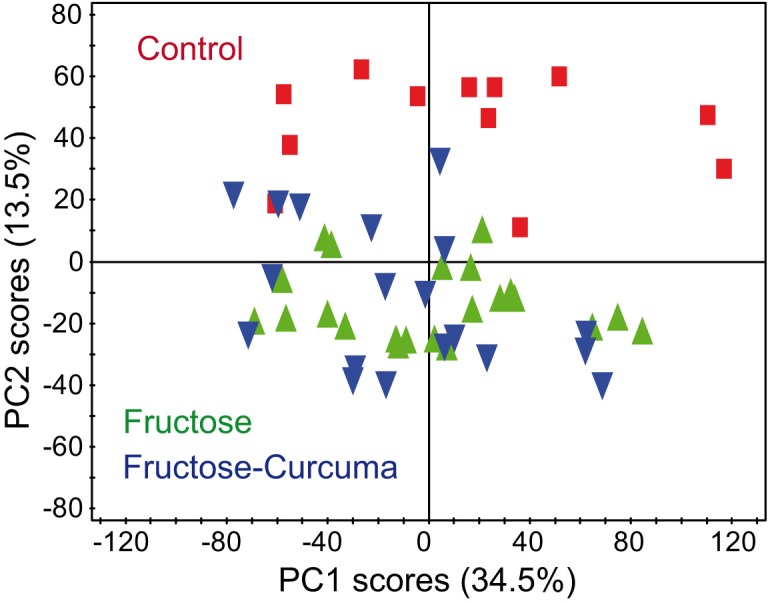
PCA score plot of the serum samples from Control, HFS and HFS+C groups.

A principal component analysis based on the correlation matrix was performed on the dataset composed of 6508 NMR variables (“buckets”) and 21 fatty acids in order to detect potential outliers and to highlight sample clustering. The first two principal components score plot explaining 34.5% and 13.5% of the total variance, respectively, is shown in Fig 2. A clear discrimination between the controls and the rats that received the diet enriched in fructose and saturated fat was visible along the PC2 axis, although this model was not capable of discriminating between HFS and HFS+C groups. We also noticed no effect of the replication of the fatty acid quantification in the X-matrix when we compared the PCA models built with or without replication (see S2 Fig).

**Fig 3:**
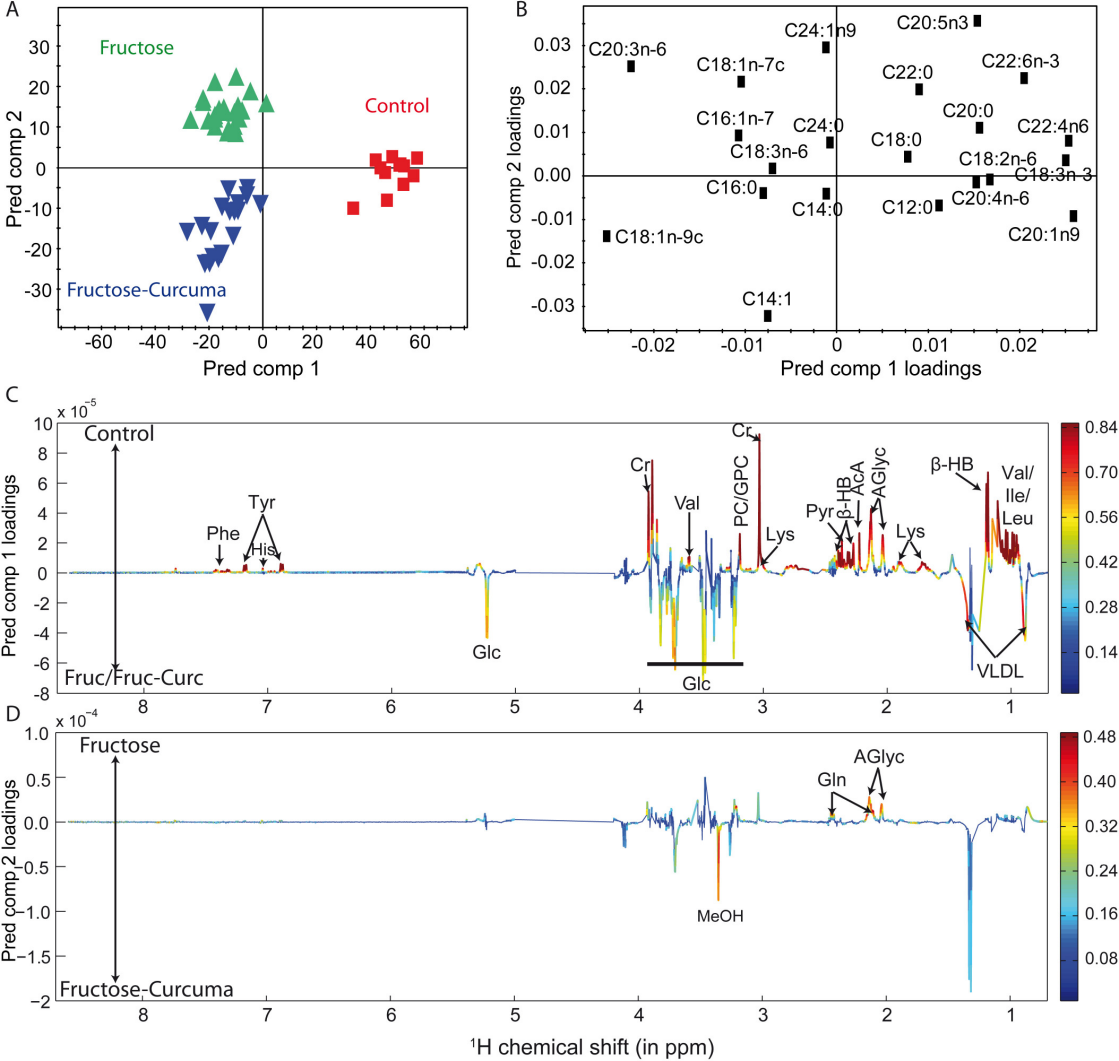
OPLS-DA score and loadings plots. (A) OPLS-DA score plot representing the 54 samples in the Tpred 1 vs Tpred 2 plane. (B, C-D) OPLSDA loadings plot representing the weights of the relative fatty acid contents and the NMR signals, respectively, along the two predictive components derived from OPLS-DA model of serum samples obtained from controls, HFS and HFS+C groups. The line variation (C, D) corresponds to model covariance derived from the mean-centered model, whereas the color map corresponds to model correlation derived from the unit-variance model.

Instead, a 2 predictive (Tpred) and 4 orthogonal components OPLS-DA model with a p-value of 1.2 10^−3^ (R2Y = 0.64, Q2Y = 0.46) enabled a clear discrimination between the three studied groups. The first predictive component was related to the diet-induced metabolic variations (Fig 3A) involving a decrease of several amino acids (phenylalanine, tyrosine, histidine, valine, lysine, isoleucine, leucine), pyruvate, acetoacetate, β-hydroxybutyrate, creatine, phosphocholine and glycerophosphocholine, alpha-linoleic (18:2n-6), alpha-linolenic (C18:3n-3), gondoic (C20:1n-9), arachidonic (C20:4n-6), adrenic (C22:4n-6), docosahexaenoic (C22:6n-3) acids associated with an increase of glucose, very low density lipoproteins (VLDL), palmitoleic (C16:1n-7), oleic (C18:1n-9) and dihomo-gamma-linolenic (C20:3n-6) acids in HFS and HFS+C groups (Fig 3B). The second predictive component reflects the curcuma-induced metabolic variations involving a decrease of glutamine, acetylated glycoproteins, eicosopentaenoic (C20:5n-3), docosahexaenoic (C22:6n-3), nervonic (C24:1n-9) acids combined with an increase of methanol, oleic (C18:1n-9) and myristoleic (C14:1n-5) acids. The Y-permutation model validated the robustness of the model and confirmed that the observed metabolic variations were not randomly induced (see S3 Fig). The relative discriminant metabolite variations are reported in Table 5.

**Table 5:** Significantly differential metabolites in the rat serum of control, HFS and HFS+C group.

metabolites	Changes in HFS	Changes in HFS+C	Changes in HFS+C
	(vs Controls)	(vs Controls)	(vs HFS)
**Acetoacetate**	↓ 1.43	↓ 1.29	
**β-hydroxybutyrate**	↓ 1.45	↓ 1.39	
**Creatine**	↓ 1.53	↓ 1.82	
**Glucose**	↑ 1.37	↑ 1.31	
**Glutamine**			↓ 1.13
**Glycoproteins (acetyl)**		↓ 1.12	↓ 1.13
**Histidine**	↓ 1.30	↓ 1.44	
**Isoleucine**	↓ 1.36	↓ 1.25	
**Leucine**	↓ 1.21	↓ 1.17	
**Lysine**	↓ 1.17	↓ 1.13	
**Methanol**		↑ 1.55	↑ 1.40
**Phenylalanine**	↓ 1.85	↓ 1.43	
**Phosphocholine/ Glycerophosphocholine**	↓ 1.21	↓ 1.25	
**Pyruvate**	↓ 1.23	↓ 1.14	
**Tyrosine**	↓ 1.68	↓ 1.77	
**Valine**	↓ 1.38	↓ 1.33	
**VLDL**	↑ 1.51	↑ 1.55	
***Fatty acids***			
**C14:1n-5 Myristoleic acid**		↑ 2.54	↑ 2.20
**C16:1n-7 Palmitoleic acid**	↑ 1.45	↑ 1.30	
**C18:1n-9 Oleic acid**	↑ 2.28	↑ 2.78	↑ 1.22
**C18:2n-6 Linoleic acid**	↓ 1.30	↓ 1.28	
**C18:3n-3 alpha Linolenic acid**	↓ 3.36	↓ 4.43	
**C20:1n-9 Gondoic acid**	↓ 4.64	↓ 3.33	
**C20:3n-6 Dihomo gamma linolenic acid**	↑ 4.18	↑ 3.29	
**C20:4n-6 Arachidonic acid**	↓ 1.24	↓ 1.30	
**C20:5n-3 Eicosopentaenoic acid**		↓ 5.55	↓ 3.69
**C22:4n-6 Adrenic acid**	↓ 3.90	↓ 7.55	
**C22:6n-3 Docosahexaenoic acid**	↓ 1.49	↓ 1.55	↓ 2.31
**C24:1n-9 Nervonic acid**			↓ 1.38

The serum metabolites that contributed significantly to the discrimination between the different diets in the OPLS-DA model from the ^1^H NMR data. ↑ (or ↓) denotes the relative increased (or decreased), followed by fold change in metabolite level (p < 0.05). The levels were calculated from relative intensities of ^1^H NMR spectra following spectral normalization.

## Discussion

### Effects of HFS and HSF+C diets on the metabolism

Our discussion will focus on the metabolic variations induced by the curcuma supplement extract. However, to put this in perspective, we first analyzed the metabolic variations common to HFS and HFS+C groups.

#### Classical tests

Interestingly, compared to HFS group, rats having received extract of curcuma tended to have a lower insulin level and higher concentration of triglycerides in the serum. These observations points towards specific but incomplete protective effects of curcumin for insulinemia and indirectly for fatty liver disease, as the most elevated triglyceride level observed in the HFS+C group may indicate a release of fat from the liver. Regulation of glucose and cholesterol, previously reported in the literature for diets with curcuma complement, was not observed [24,25]. This is most likely due to the much lower dosage (1 mg/kg body weight/day) of curcuminoids that we used, 1 to 2 order of magnitude less compared to previous studies, which led to no detectable curcumin in the serum [24,26]. Note that our dosage was selected to correspond to the habitual intake of curcuminoids in the Asian population as recommended in the joint report of the Food and Agriculture Organization and the World Health Organization on food additives [27].

#### Oxidative stress

Our results (Table 3) associated HFS groups rats with an increase in lipid peroxidation and a decrease in TAC in serum, suggesting increased in ROS production and/or a weakening of the antioxidant system defenses. Fructose-induced hyperglycemia and high plasma levels of triglycerides could promote increasing generation of ROS and reducing the antioxidant defense status [28].

The oral administration of curcuma extract did not counteract the oxidative stress induced by the HFS diet, in contrast to previous studies, which have shown that CL powder by itself, and its major bioactive component curcumin, would protect against oxidative stress thanks to a radical-trapping ability as chain-breaking antioxidant [29]. As seen above, the discrepancy may be explained by the dose of curcuminoids and the composition of diet used here.

### NMR and GC/MS discrimination between Control and HFS/HFS+C groups

Table 5 reports the observed metabolite modulations with respect to the diets of our animal model. High-level of glucose and VLDL are well-characterized effects of a HFS diet [6,22]. The increase in the availability of gluconeogenic substrates have been suggested to be the major cause of fasting hyperglycemia in HFS-fed rats [30]. An increase in the production of VLDL in animal of high-fructose feeding may be driven by IR [31], increased triglyceride flux and hepatic inflammation [32], and decreased clearance [33]. The use of choline or phosphatidylcholine for VLDL secretion could explain the depletion of phosphocholine and glycerophosphocholine [34]. Furthermore, this depletion also suggests a possible involvement of gut microbiota, which in high fat diet-induced IR mice was found to convert dietary choline into methylamines with an increased level of methylamines urinary excretion, thereby reducing the biodisponibility of choline [35].

Considering that the blood samples were drawn after a fasting period, it is not surprising that the ^1^H NMR spectrum comparison between controls and HFS groups revealed a substantial perturbation of key metabolites related to energy production. Indeed, HFS rat groups had lower ketone bodies, pyruvate, creatine, and amino acids. Pyruvate, creatine and glycoproteins were reported in a recent NMR metabolomics studies to be more abundant in lean vs. obese pigs sera [36]. The level of decreasing pyruvate in HFS groups suggests an enhancement of the lipogenesis observed in fructose consumption [37].

The lower level of creatine in the HFS and HFS+C groups may be linked to the depletion of choline, as the deficiency of this latter increases the utilization of S-adenosylmethionine for phosphatidylcholine synthesis, making it less available for the synthesis of creatine [38]. Moreover, creatine has an antioxidant activity and its reduction could be related to the oxidative stress measured in HFS groups [39].

More abundant acetoacetate and β-hydroxybutyrate in the control serum at concentration detectable by ^1^H NMR is not surprising due to the several hours of fasting imposed before sacrifice. While the role of ketone bodies (KB) besides being energy carriers is still being investigated [40], the reduced concentration of KB in HFS groups correlates well with the inhibition of ketogenesis by insulinemia [41] and with the higher glucose concentration observed in HFS groups. Given that IR suppresses hepatic FA catabolism and stimulates hepatic lipogenesis and FA esterification [42], HFS diet that leads to IR may cause a greater fractional esterification of FA and a lower beta-oxidation of KB [43].

We also observed a lower relative concentration of several amino acids: phenylalanine, tyrosine, histidine, lysine, and the branched chain amino acids (BCAA) isoleucine, leucine, valine in both HFS groups compared to controls. This is consistent with previously reported studies [13,44], suggesting a perturbation of the energy metabolism. Under hyperinsulinemia conditions or in the presence of FA-induced IR, measured plasma amino acid concentrations have been observed to decline, suggesting lower rates of protein breakdown and protein synthesis [45,46]. Furthermore, there are conflicting data concerning the role of BCAA: a study reported that supplementation of a high fat diet in rats with BCAA contributed to development of IR [47], while another one has demonstrated that higher BCAA intake in humans is inversely associated with prevalence of overweight and obesity [48]. Moreover, most of these amino acids are gluconeogenic and may contribute to elevated rate of gluconeogenesis as reported in high fructose diet [30], the exception being leucine and lysine, which play a protective role in the IR, improving insulin sensitivity [49] and inhibiting protein glycation, respectively [50].

Out of the twenty-one fatty acids quantified in this study, twelve have their concentration altered significantly between controls and HFS rats. The serum FA profile in HFS-fed rats was characterized by a higher proportion of MUFA and by a lower proportion of PUFA, as already observed in our previous study [22]. This behavior corresponds in general to a higher oxidation activity as detected in the HFS groups. More specifically, an increased activity of Δ9D observed in HFS group, coupled to the increase in triglycerides could represent an initial cellular defense against lipotoxicity in response to acute HFS diet, for the sequestering of palmitic acid into triglycerides [51]. The decrease of n-3 PUFA, alpha-linolenic acid (18:3 n-3), EPA (20:5n-3) and DHA (22:6 n-3) induced by the HFS diet could be involved in early processes that lead to IR [52].

### Specific effects of curcuma dietary supplement on the metabolism

According to the OPLS-DA loadings (Fig 3B and 3D), alterations of some FAs, an increase of methanol and a decrease of glutamine and glycoproteins were observed in the HFS+C group compared to the HFS one.

#### Fatty acids

The presence of curcuminoids in the diet induced some noticeable alterations of the FA metabolism with respect to the HFS diet: a further increase in some MUFA (myristoleic acid, oleic acid, nervonic acid) and a further decrease in eicosopentaenoic and docosahexaenoic acid suggesting that the metabolism of the n-3 PUFAs was affected. Curcumin is a non-specific and non-competitive inhibitor of the desaturases in rat liver microsomes [53], which could explain our results. There is a consensus on the existence of competition among fatty acids of the n-3 and n-6 families for desaturation and chain elongation, 18:3 n-3 being a better substrate for Δ6 desaturase than 18:2 n-6 [54]. The presence and increase of 14:1n-5 (myristoleic acid) provided evidence that 14:0 (myristic acid) was also converted by stearoyl-CoA desaturase. Associated with the increase of 18:1n-9, it suggests a stimulation of stearoyl-CoA desaturase activity by curcumin. Thus, as reported above, the raise in 18:1n-9 could lead to the accumulation of triglycerides that rescues C16:0-induced apoptosis by channelling C16:0 into triglycerides and away from pathways introducing to apoptosis [52]. The level of nervonic acid (24:1n-9) was significantly depressed by addition of CL extract to the diet. This FA is normally present only in sphingomyelin, a major component located mostly in the outer layer of the plasma membrane and plays a role in maintaining the membrane in a more rigid state [55]. In overweight and obese female erythrocytes membranes, 24:1n-9 was found increased and correlated with decreased membrane fluidity [56]. Given that the IR and the decrease of membrane fluidity are associated [57], curcumin could offer protection from the rigidifying of the membrane by decreasing the level of 24:1n-9 in acting on the elongation steps of its synthesis. Further experiments are required to elucidate this finding.

#### Methanol

Intriguingly, we observed a higher level of methanol in HFS+C group compared to HFS group. Although generally endogenously produced from gut microbiota, methanol is more likely to be generated here as a product of mammalian intermediary metabolism [58]. The methanol formed would be oxidized sequentially to formaldehyde, then to formic acid and finally to carbon dioxide. This occurs mainly in the liver, mediated by alcohol dehydrogenase (ADH) and aldehyde dehydrogenase (ALDH) [59]. In rats, catalase is also a significant pathway of methanol metabolism [60]. Fructose decreases catalase-dependent alcohol oxidation due to the inhibition of H_2_O_2_ generation via peroxisomal beta-oxidation of fatty acids by decreasing ATP and the NAD/NADH ratio [61]. Thus, in HFS diets, the oxidation of methanol should be mediated predominantly via ADH. In this work, the curcuma-free oral vehicle and the hydroalcoholic extract of curcuma (methanol-free) administered in aqueous suspension daily to the rats contained 2% of ethanol, which is able to inhibit the oxidation of methanol by ADH [62]. Indeed, this latter has a better affinity and specificity for ethanol than for methanol [63], thus ethanol is preferentially metabolised by ADH. In addition, it has been shown that fructose increases the rate of ethanol metabolism in both animals and man [64,65]. Since HFS and HFS+C groups received the same amount of ethanol, the higher level of methanol observed in the HFS+C group can stem from the curcuminoids present in the turmeric extract. Interestingly, previous studies have shown that curcumin is a substrate of ADH and a preparation of curcumin impaired the ethanol oxidation via ADH in humans [66]. Thus, the increase in methanol level observed in HFS+C group might be explained by an inhibition of methanol (alcohol) elimination in the liver with a concomitant constant endogenous production.

#### Glutamine and glycoproteins

An increased level of glutamine and glycoproteins were observed in the HFS group compared to HFS+C one. This suggests alterations in glutamine metabolism as observed in type II diabetics [67] and a possible activation of the hexosamine biosynthesis pathway (HBP). The HBP is a minor branch from glycolysis pathway from which fructose-6-phosphate (F-6-P) amidotransferase (GFAT) forms glucosamine-6-phosphate (GlcN-6-P) [68]. Subsequent enzymatic reactions metabolize GlcN-6-P to UDP-N-acetylglucosamine (UDP-GlcNAc), the major end product of the HBP [69]. Previous studies have demonstrated that HBP can play a role in IR, given that many proteins involved in insulin signal transduction (as insulin receptor substrate, IRS) or in glucose transporter (GLUT) are subject to O-GlcNAcylation [70]. Moreover, glutamine is a key inducer of HBP flux by providing the amide group for the formation of hexosamines and it is required in desensitization of insulin-responsive glucose transport system [71]. Thus, the hyperglycemia seen in HFS group could increase the glucose availability through the HBP leading to accumulation of glycoproteins and leading to IR. On the other hand, despite the extract of curcuma used here not normalising glycemia, a lower level of glycoproteins was observed in the HFS+C group. This is in agreement with a study showing that oral administration of tetrahydrocurcumin (a metabolite of curcumin) to diabetic rats decreased the level of plasma glycoproteins [72].

## Conclusion

A view of the metabolic changes in an animal model fed with high fructose and saturated fatty acid diet, as long as the effects of integration with the nutritional supplement *Curcuma longa* has been provided by NMR metabolomics and GC-MS lipidomics of the serum. While the observed and expected, diet-induced, metabolic disorders (insulin resistance) could not be countered by the nutritional supplement, elements have been discerned of a possible localized protection, notably at the liver level. Indeed, while perturbation of several energy-related metabolites (amino acids, ketonic bodies, creatine, pyruvate, glucose, VLDL) have been observed in both animals fed with the high fructose and saturated fatty acid diets, despite *Curcuma longa* dietary supplementation, specific biomarkers could be associated to this latter setup. Particularly, a depression of the alcohol oxidation, detected through an enhanced concentration of methanol, and a possible activation of the hexosamine biosynthesis pathway, consistent with a reduction of glutamine and glycoprotein levels. Both these two effects point towards a modified liver function. Further biomarkers of the curcuminoid enriched diet were a significant alteration of the concentration of long chain unsaturated fatty acids: C20:5n-3, C22:6n-3 and C24:1n-9. The variation in the concentration in this latter is a specific effect of the nutritional supplement, as it is not observed in the high fructose and saturated fatty acid diet groups, and is suggestive of a cellular enhanced response to the membrane rigidification associated to insulin resistance. All of these points to possible specific and local, rather than systemic, protective effects of *Curcuma longa*, and further studies are underway to clarify this issue.

## Supporting Information

S1 Fig ^1^H NOESY NMR spectrum of serum sample from rat fed with a HFS diet supplemented with curcuma extract. Assignments: 1, lipids; 2, isoleucine; 3, leucine; 4, valine; 5, propylene glycol; 6, β-hydroxybutyrate; 7, lipids; 8, lactate; 9, alanine; 10, lipids; 11, lysine; 12, lipids; 13, acetate; 14, glycoproteins (acetyl); 15, acetoacetate; 16, unknown; 17, glutamate; 18, pyruvate; 19, glutamine; 20, citrate; 21, lipids; 22, creatine; 23, choline; 24, phosphocholine/glycerophosphocholine; 25, methanol; 26, alpha-glucose and beta-glucose; 27, lipids; 28, cytidine; 29, tyrosine; 30, histidine; 31, phenylalanine; 32, formate.
(PDF)

S2 Fig PC1 vs PC2 score plot of 28 serum samples. For this analysis, one aliquot per sample was considered.
(PDF)

S3 Fig Validation plot. Model validation resulting from 999 permutations, demonstrating the model robustness, because model R2 and Q2 values were significantly higher than random model ones.
(PDF)

S1 Table Assignment of ^1^H and ^13^C NMR signals of metabolites. (PDF)
