# Supplementary material for: Metabolomic and Lipidomic Analysis of Serum Samples following Curcuma longa Extract Supplementation in High-Fructose and Saturated Fat Fed Rats
Source: PLoS One. 2015 Aug 19;10(8):e0135948. doi: 10.1371/journal.pone.0135948 (PMC4545834; doi:10.1371/journal.pone.0135948)
Supplement: S1 Fig — Assignments: 1, lipids; 2, isoleucine; 3, leucine; 4, valine; 5, propylene glycol; 6, β-hydroxybutyrate; 7, lipids; 8, lactate; 9, alanine; 10, lipids; 11, lysine; 12, lipids; 13, acetate; 14, glycoproteins (acetyl); 15, acetoacetate; 16, unknown; 17, glutamate; 18, pyruvate; 19, glutamine; 20, citrate; 21, lipids; 22, creatine; 23, choline; 24, phosphocholine/glycerophosphocholine; 25, methanol; 26, alpha-glucose and beta-glucose; 27, lipids; 28, cytidine; 29, tyrosine; 30, histidine; 31, phenylalanine; 32, formate. (PDF) [file pone.0135948.s001.pdf]

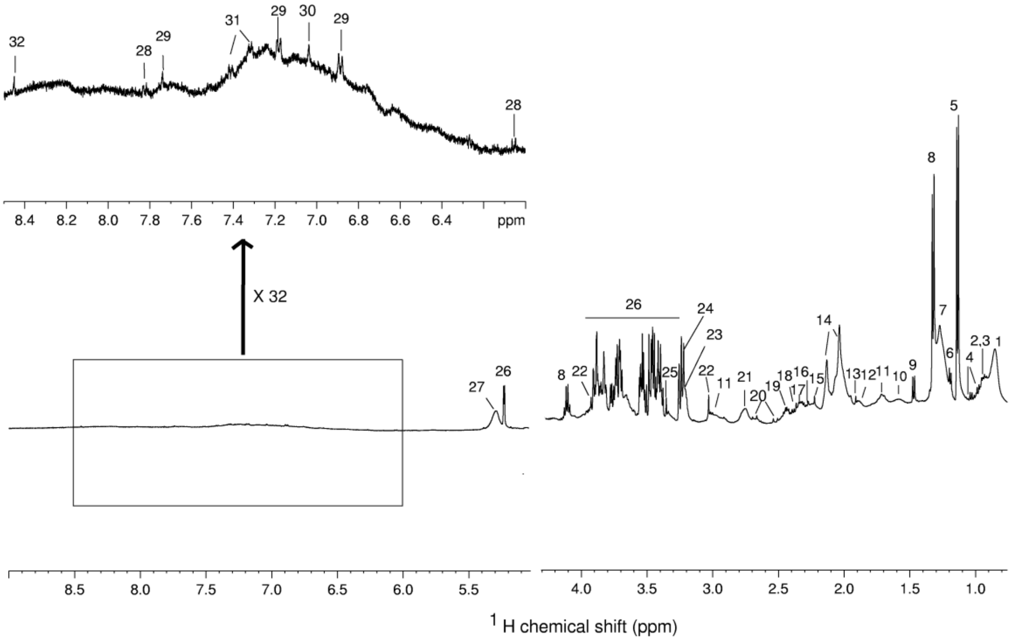

S1 Fig. ^{1}H NOESY NMR spectrum of serum sample from rat fed with a HFS diet supplemented with curcuma extract.
