# Supplementary figures and images for: Metabolomic and Lipidomic Analysis of Serum Samples following Curcuma longa Extract Supplementation in High-Fructose and Saturated Fat Fed Rats
Source: PLoS One. 2015 Aug 19;10(8):e0135948. doi: 10.1371/journal.pone.0135948 (PMC4545834; doi:10.1371/journal.pone.0135948)

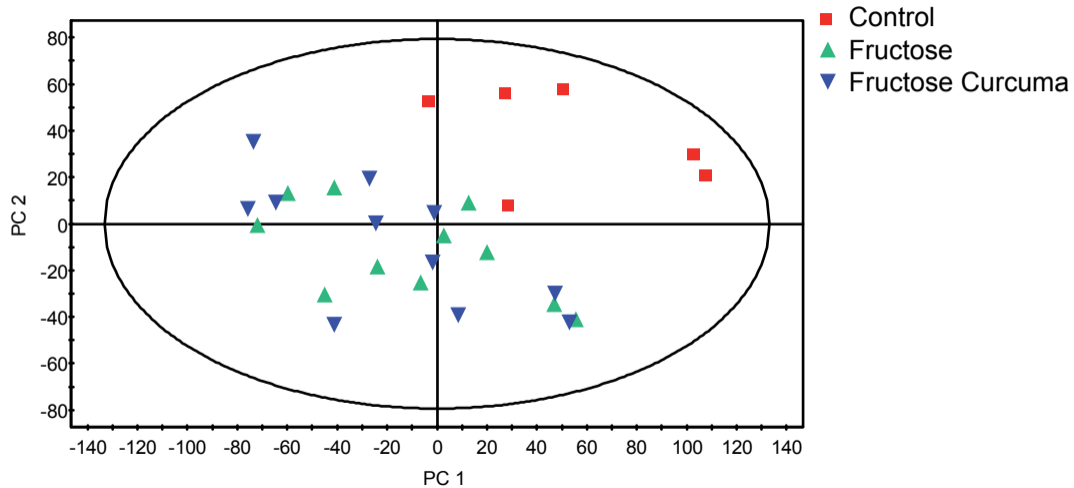

Supplement: S2 Fig — For this analysis, one aliquot per sample was considered. (PDF) [file pone.0135948.s002.pdf]

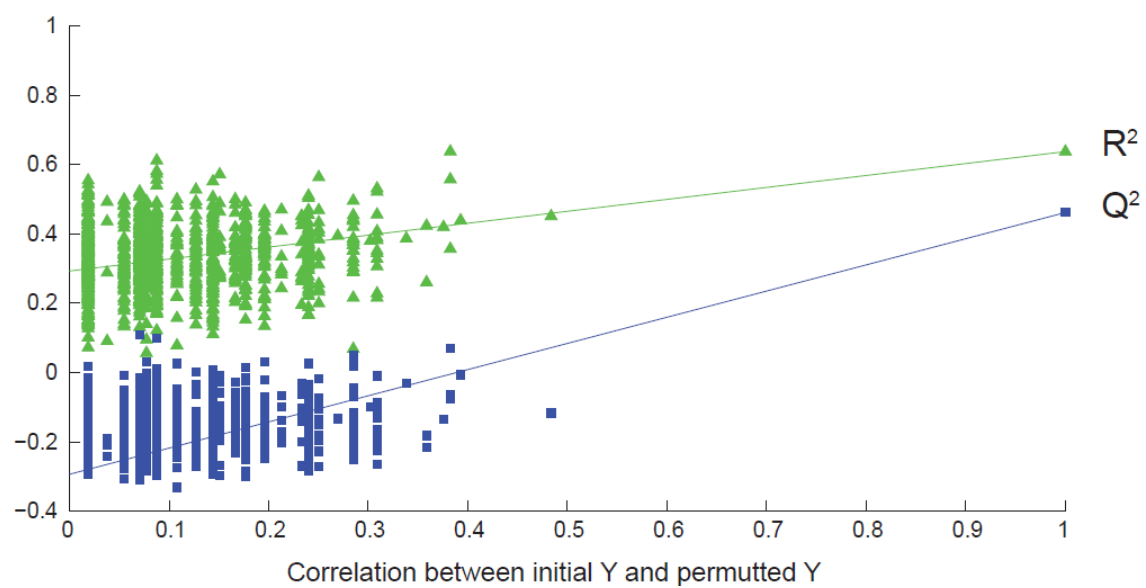

**S3 Fig. Model validation.**

Supplement: S3 Fig — Model validation resulting from 999 permutations, demonstrating the model robustness, because model R2 and Q2 values were significantly higher than random model ones. (PDF) [file pone.0135948.s003.pdf]
