# Supplementary material for: Metabolomic and Lipidomic Analysis of Serum Samples following Curcuma longa Extract Supplementation in High-Fructose and Saturated Fat Fed Rats
Source: PLoS One. 2015 Aug 19;10(8):e0135948. doi: 10.1371/journal.pone.0135948 (PMC4545834; doi:10.1371/journal.pone.0135948)
Supplement: S1 Table — (PDF) [file pone.0135948.s004.pdf]

S1 Table. Assignments of metabolites ^{1}H and ^{13}C NMR.

compound	^{1}H chemical shift (in ppm)	multiplicity	^{13}C chemical shift (in ppm)	assignment
Acetate	1.91	s	24.3	CH_3
Acetoacetate	2.23	s	36.5	CH_3
Alanine	3.77<br>1.47	q<br>d	53.7<br>18.9	CH<br>CH_3
Allantoin	5.38	s		CH
Alpha-glucose	5.23<br>3.83<br>3.82<br>3.70<br>3.53<br>3.41	d<br>unresolved<br>unresolved<br>unresolved<br>unresolved<br>unresolved	95.0<br>63.4<br>74.3<br>72.7<br>74.4<br>78.6	H1<br>half CH_2-C6<br>H5<br>H3<br>H2<br>H4
Beta-glucose	4.64<br>3.90<br>3.72<br>3.47<br>3.40<br>3.24	unresolved<br>unresolved<br>unresolved<br>unresolved<br>unresolved<br>unresolved	98.8<br>63.6<br>63.4<br>78.6<br>72.5<br>77.0	H1<br>half CH_2-C6<br>half CH_2-C6<br>H5<br>H4<br>H2
Beta-hydroxybutyrate	4.14<br>2.39<br>2.30<br>1.19	unresolved<br>m<br>m<br>d	 49.2<br>49.2<br>24.4	betaCH<br>half alphaCH_2<br>half alphaCH_2<br>gammaCH_3
Betaine	3.26<br>3.89	s<br>s	56.3	CH_3<br>CH_2
Choline	4.06<br>3.51<br>3.19	m<br>m<br>s	58.0<br>69.5	alphaCH_2<br>betaCH_2<br>CH_3
Citrate	2.68<br>2.53	d<br>d	47.3<br>47.3	CH_2(i)<br>CH_2(ii)
Creatine	3.93<br>3.04	s<br>s	 39.5	CH_3<br>CH_2
Cytidine<br>(provisional)	7.82<br>6.04	d		
Lipid	2.23<br>2.07<br>2.01<br>1.58<br><br>1.30<br>0.93<br>0.89	broad<br>broad<br>broad<br>broad<br><br>broad<br>broad<br>broad	34.2<br><br><br>25.0<br><br><br>20.9<br>25.6	CH_2CO<br>CH_2<br>$CH_2C=C$<br>CH_3CH_2<br>(CH_2)<br>CH_2<br>CH_3CH_2<br>CH_3CH_2
Lipid (VLDL)	1.29<br>0.87	broad<br>broad	27.2<br>16.8	$CH_2CH_2CH_2CO$<br>$CH_3CH_2CH_2C=$

Formate	8.44	s		CH
Fumarate	6.51	s - broad		CH
Glutamate	2.34<br>2.03	m<br>m	33.9<br>29.7	gammaCH_2<br>betaCH_2
Glutamine	3.74<br>2.44<br>2.12	 m<br>m	57.6<br>34.1<br>27.1	alphaCH<br>gammaCH_2<br>betaCH_2
Glyceryl of lipids	5.20<br>4.26<br>4.06	broad<br>broad<br>broad		CHOCOR<br>CH_2OCOR<br>CH_2OCOR
Glycine	3.55	s	44.8	CH_2
Glycoproteins (O-acetyl)<br>(N-acetyl)	2.15<br><br>2.04	s - broad<br><br>s - broad	23.2<br><br>25.0	$OCOCH_3$<br><br>$NHCOCH_3$
Histidine	7.73<br>7.02	s<br>s		H2<br>H5
Isoleucine	1.97<br>1.47<br>1.00<br>0.93	broad<br>broad<br>d<br>unresolved	 <br>17.3 	betaCH_2<br>half gammaCH_2<br>gammaCH_3<br>deltaCH_3
Lactate	4.11<br>1.32	q<br>d	71.5<br>22.9	CH_3<br>CH
Leucine	3.67<br>1.71<br><br>0.94	dd<br>m<br><br>d	56.3<br>42.7<br><br>20.9	alphaCH<br>CH_2 and<br>gammaCH<br>delta-CH_3
Lysine	3.73<br>3.03<br>1.89<br>1.70<br>1.47	unresolved<br>m<br>m<br>m<br>m	57.3<br>39.2<br>33.1<br>29.6<br>25.4	alphaCH<br>epsilonCH_3<br>betaCH_2<br>deltaCH_2<br>gammaCH_2
Malic acid<br>(provisional)	4.30<br>2.36	dd<br>dd	 44.3	CH<br>CH_2
Methanol	3.35	s	52.0	CH_3
Methionine	2.63<br>2.12	m<br>m	31.7<br>16.8	SCH_2<br>betaCH_2
Myo-inositol	4.08<br>3.61	unresolved<br>unresolved	77.8<br>72.8	H2<br>H4, H6
Phenylalanine	7.41<br>7.32	m<br>m		H3, H5<br>H2, H6
Phosphocholine/<br>Glycerophosphocholine	4.32<br>3.67<br>3.21	m<br>m<br>s	62.7<br>68.3<br>56.8	alphaCH_2<br>betaCH_2<br>CH_3

Proline	3.33<br>2.34<br>2.06	unresolved<br>unresolved<br>unresolved	45.3<br>33.9<br>29.7	Half deltaCH_2<br>Half beta-CH_2<br>Half beta-CH_2
Propylene glycol	3.87<br>3.53<br>3.43<br>1.13	m<br>dd<br>dd<br>d	70.8<br>69.8<br>69.6<br>21.6	CH<br>CH_2(i)<br>CH_2(ii)<br>CH_3
Pyruvate	2.36	s	29.2	CH_3
Threonine	4.21<br>3.59	m<br>d	68.6<br>63.1	betaCH<br>alphaCH
Thymidine<br>(provisional)	6.26<br>4.43<br>2.41	t<br>unresolved<br>unresolved		(2)CH<br>(4)CH<br>(3)CH_2
Tyrosine	7.18<br>6.88	d<br>d		oCH<br>mCH
Valine	3.58<br>2.27<br>1.03<br>0.98	unresolved<br>m<br>d<br>d	63.1<br>31.1<br>20.9<br>18.5	alphaCH_2<br>betaCH<br>Gamma-CH_3<br>Gamma′-CH_3
VLDL	0.87	broad	16.8	CH_3CH_2
Unknown 1	4.32<br>1.38		 22.8	
Unknown 2	3.66<br>2.67<br>1.71		31.5<br>42.7<br>42.7	
Unknown 3	3.34<br>2.46	broad<br>broad	48.6<br>34.2	
Unknown 4	3.00<br>2.43		 33.7	
Unknown 5	2.75<br>1.78		42.5<br>42.3	
Unknown 6	7.89<br>7.43			
Unknown 7	7.82<br>6.05			
Unknown 8	7.77<br>7.46			
Unknown 9	7.11<br>6.81			

Unsaturated lipid	5.32<br>5.29<br>2.76<br>2.03<br>1.30	broad<br>broad<br>broad<br>broad<br>broad	132.8<br>130.2<br>28.2<br>26.0<br>32.0	CH=CH<br>CH=CH<br>CH2<br>CH_2CH=<br>CH_2
Uracil	7.52<br>5.79	broad<br>broad		H6<br>H5
Uridine	7.86<br>5.89	broad<br>broad		H11<br>H10
